# Lipoprotein-Associated Phospholipase A2 Is a Risk Factor for Patients With Parkinson’s Disease

**DOI:** 10.3389/fnins.2021.633022

**Published:** 2021-04-20

**Authors:** Zubo Wu, Suyuan Wu, Tao Liang, Lin Wang

**Affiliations:** ^1^Department of Pediatrics, Union Hospital, Tongji Medical College, Huazhong University of Science and Technology, Wuhan, China; ^2^Department of Clinical Laboratory, Union Hospital, Tongji Medical College, Huazhong University of Science and Technology, Wuhan, China

**Keywords:** Lp-PLA2, Parkinson’s disease, risk factor, neuroinflammation, neurodegenerative diseases

## Abstract

**Objective:**

To explore the association between lipoprotein-related phospholipase A2 (Lp-PLA2) and the risk of Parkinson’s disease (PD).

**Methods:**

A case-control study involving 58 hospitalized PD patients and 60 healthy controls was carried out. Serum Lp-PLA2 level was detected. According to the disease course and severity, PD patients were subdivided to analyze the clinical value of Lp-PLA2. Relationship between Lp-PLA2 and PD risk was analyzed by logistic regression. Diagnostic value of Lp-PLA2 in PD patients was investigated using receiver’s operator characteristic curves.

**Results:**

Lp-PLA2 level was significantly higher in the PD patients compared with the controls, and was significantly and positively correlated with the Hoehn-Yahr (H&Y) stage. The serum Lp-PLA2 level and H&Y stage of PD patients with a longer disease course were significantly higher than those with a shorter disease course. PD patients with milder conditions had significantly lower serum Lp-PLA2 levels than patients with severe conditions. Multivariable logistic regression analysis indicated higher Lp-PLA2 level was an independent risk factor of PD patients. Moreover, the area under the curve for Lp-PLA2 was 0.703, which was between those of homocysteine and serum amylase A.

**Conclusion:**

To our knowledge, this is the first study to show that increased level of Lp-PLA2 is associated with the risk of PD. Lp-PLA2 may be used for early detection of PD, and provides an effective intervention target for clinical treatment of PD.

## Introduction

Parkinson’s disease (PD) is a common neurodegenerative disorder that occurs mostly in middle-aged and elderly people. Its main pathological features are the formation of Lewy bodies in the cytoplasm of neurons, gliosis, and loss of dopaminergic neurons in the substantia nigra striatum (Raza et al., 2019). The clinical manifestations of PD are mainly static tremor, muscle stiffness, slow movement, and abnormal posture and gait. At the same time, PD patients also show various non-motor symptoms, such as depression, cognitive dysfunction, and sleep disturbance (Sveinbjornsdottir, 2016). Although many theories have attempted to explain the etiology of PD, the exact pathogenesis is not yet fully understood. In addition to the widely-studied pathological mechanisms of mitochondrial dysfunction (Bose and Beal, 2016), oxidative stress (Dias et al., 2013), and abnormal protein degradation (Ebrahimi-Fakhari et al., 2012), growing evidences from human samples and animal models show that inflammation plays a key role in the pathogenesis of PD (Dzamko et al., 2015; Caggiu et al., 2019; Chen et al., 2019).

Neuroinflammation is a double-edged sword. It helps the recovery of damaged neurons, but can also induce or aggravate the neurodegeneration of the central nervous system (CNS; Kennedy, 2015). Current researches show that inflammation has become a common cause of many neurodegenerative diseases, such as Alzheimer’s disease (AD; Sinyor et al., 2020), dementia (Peila and Launer, 2006), amyotrophic lateral sclerosis (McCauley and Baloh, 2019), and Huntington’s disease (Pac et al., 2020). Studies confirm that central inflammation and peripheral inflammation jointly participate in the occurrence and development of PD (Su and Federoff, 2014; Kustrimovic et al., 2019). One convincing evidence is that the use of non-steroidal anti-inflammatory drugs can decrease the risk of PD (Esposito et al., 2007). Postmortem analysis of PD patients and experimental animal studies shows that high levels of pro-inflammatory factors and microglia activation are common features of PD brain tissues (Kannarkat et al., 2013; Joers et al., 2017). In addition, α-synuclein (α-syn) misfolds and forms toxic bodies in the cytoplasm, which is an important pathogenic factor of PD (Kalia and Kalia, 2015). Studies demonstrate that α-syn can induce the activation of microglia in the brain, leading to cerebral inflammation (Rocha et al., 2018). In addition to the inflammatory response of the CNS, invasion and accumulation of peripheral immune cells also occur in the brains of PD patients. The blood-brain barrier (BBB), as an important gateway between the CNS and the peripheral circulatory system, is a selective biological barrier (Stamatovic et al., 2008). Under normal physiological conditions, the BBB has extremely low permeability to immune cells and cytokines in the peripheral system (Younger, 2019). However, when the BBB is damaged, especially after the structural destruction of brain capillaries, it can cause peripheral inflammation to cross the BBB and trigger the activation of inflammatory cells in the PD brain (Pan and Nicolazzo, 2018; Karakatsani et al., 2019). Therefore, maintaining the normal structure and function of the BBB is essential for PD.

Lipoprotein-associated phospholipase A2 (Lp-PLA2), also known as platelet-activating factor acetylhydrolase, is a calcium-independent lipase mainly produced by monocytes and macrophages. Lp-PLA2 can hydrolyze the oxidized phospholipids in low-density lipoproteins (LDLs) through its enzymatic activity and release pro-inflammatory substances (e.g., oxidized free fatty acids and lysolecithin). Consequently, it can trigger a series of inflammatory cascades, such as up-regulating endothelial cell adhesion molecules and cytokines, inducing the chemotaxis of leukocytes and monocytes, and promoting their entry into the inner membranes of blood vessel walls (McConnell and Hoefner, 2006; Huang et al., 2020). As a high-risk factor for atherosclerotic vascular disease and cardiovascular disease, Lp-PLA2 has received more and more attention. Recent studies have found that the level of Lp-PLA2 is not only related to the formation of lipid atherosclerotic plaques, ischemic stroke and vascular inflammation, but also to the occurrence of coronary heart disease (Bonnefont-Rousselot, 2016; Qiao et al., 2020; Qin et al., 2020). Lipid metabolism plays an important regulatory role in the occurrence and development of cardiovascular and cerebrovascular diseases (Esenwa and Elkind, 2016; Zhang et al., 2020). Some scholars have found that in patients with CNS diseases or injuries, there are abnormal lipid metabolisms in their bodies, and Lp-PLA2, as a member of the phospholipase superfamily, is even more involved in this process (Adibhatla and Hatcher, 2008). Most of the existing studies of Lp-PLA2 focus on cardiovascular diseases, vascular inflammatory diseases, and ischemic stroke (Ikonomidis et al., 2014; Lin et al., 2015; Wang et al., 2019). However, so far, there have been no reports on the relationship between the Lp-PLA2 level and PD, and the clinical value of Lp-PLA2 in PD patients is unclear. Therefore, we conducted this case-control study to investigate whether LP-PLA2 can be used as a high-risk early warning factor for PD, and to provide a basis for more accurate early identification and treatment of PD.

## Subjects and Methods

### Subjects

A total of 58 hospitalized PD patients from the Department of Neurology, Tongji Medical College Affiliated Union Hospital, Huazhong University of Science and Technology between March 2017 and June 2018 were recruited. There were 29 males and 29 females, and they were aged from 33 to 84 years, with an average of 65.24 ± 10.18 years. The course of illness ranged from 1 month to 16 years, with an average of 3 years. All cases were diagnosed by specialists according to typical clinical symptoms and imaging examinations, and in accordance with the diagnostic criteria of PD in the UK Brain Bank (Hughes et al., 2001). The exclusion criteria are as follows: (1) Parkinson’s symptoms caused by brain trauma, encephalitis, and drugs; (2) essential tremor and multiple system atrophy; (3) PD with tumors, severe infection of the whole body or CNS; (4) PD with severe diseases of the heart, liver and kidney. According to Hoehn-Yahr (H&Y) stage standard (Goetz et al., 2004), PD patients were divided into a mild PD group (*n* = 41, H&Y stage 1–2.5; phase I and II) and a moderate to severe PD group (*n* = 17, H&Y stage ≧ 2.5; phase III, IV, and V). Besides, PD patients were subdivided by the course of disease into a <5 years group (41 cases) and a ≧5 years group (17 cases). The age- and gender-matched control group consisted of 60 healthy subjects (33 males/27 females, median age 65.82 ± 4.86 years), who were recruited from the medical examination center in our hospital and had no evidence of cerebrovascular or inflammatory disease. All subjects were Chinese Han from the same area in Middle China and all gave informed consent. Our study was approved by the ethics committee at Tongji Medical College, Huazhong University of Science and Technology.

### Collection of Data

After PD patients were admitted to the hospital, their demographic data were obtained in time, including age, gender, disease course, past history, family history, and smoking and drinking habits. Clinical characteristic parameters of PD patients were also collected, such as diastolic blood pressure (DBP), systolic blood pressure (SBP), dyslipidemia, hypertension, diabetes, heart rate, respiratory rate, and body temperature. Patients with a history of hypertension or SBP ≧ 140 mmHg or DBP ≧ 90 mmHg at rest were all diagnosed as hypertension. Diabetes mellitus was diagnosed if the patient was being treated with antidiabetic medications or insulin therapy or had a fasting blood glucose level ≧ 7.0 mmol/L. We also collected other laboratory parameters upon admission, including alanine aminotransfease (ALT), aspartate aminotransferase (AST), alkaline phosphatase (ALP), glutamyl transpeptidase (GGT), urea nitrogen (BUN), creatinine (Cr), uric acid (UA), cystatin C (Cys C), fasting blood glucose (Glu), cholesterol (TC), triglycerides (TG), high-density lipoprotein (HDL), LDL, serum amylase A (SAA), homocysteine (HCY), and other blood coagulation related indicators.

### Preparation of Blood Samples

Within 24 h of admission, blood samples were collected with BD yellow tubes from peripheral veins of PD patients who had fasted for 12 h. The blood samples were centrifuged at 3,000 rpm and room temperature for 10 min to separate serum. Then the serums were immediately transferred to eppendorf tubes, labeled and stored in a refrigerator at −80°C until the detection of biochemical factors.

### Measurement of Lp-PLA2

Serum Lp-PLA2 mass was measured with a latex-enhanced scatter immunoturbidimeter (NORMAN-2, Nanjing Norman Biotechnology Co., Ltd.). Commercial Lp-PLA2 kits were also provided by NORMAN. All operations were conducted in accordance with the reagent instructions. Strict calibration and quality control procedures were also performed.

### Statistical Analysis

SPSS 22.0 (IBM Co., Armonk, NY, United States) was used for statistical analysis. Normality data was expressed as mean ± standard deviation (SD), and statistically analyzed among groups by *t* test. Non-normally distributed data were expressed as [median (*P*50), 25th to 75th percentile (*P*25∼*P*75)] and compared among groups using *Mann–Whitney U* test. Relationship between any two variables was analyzed using Spearman correlation. The risk factors for PD were determined by logistic regression analysis. After analyzing the difference between the PD group and the normal control group, we chose to incorporate those significant indicators into the logistic regression analysis model. The diagnostic value of Lp-PLA2 for PD was evaluated by calculating the area under receiver’s operator characteristic (ROC) curve (AUC). In the two-tailed test, *P* < 0.05 was considered statistically significant.

## Results

### Demographic and Clinical Characteristics of PD Patients and Healthy Controls

The demographic and clinical characteristics of all participants were shown in Table 1. No significant between-group difference was found in gender, age, ALP, Cr, Glu, TG, or HDL (*P* > 0.05). The results showed that the levels of Cys C, SAA, HCY, and Lp-PLA2 in the PD group were significantly increased, while the levels of ALT, AST, BUN, TC, and LDL in this group were significantly lower than those in the control group (all *P* < 0.05),which remained significant after Bonferroni correction. However, after Bonferroni correction, UA no longer had a significant difference.

**TABLE 1:** Demographic and clinical characteristics.

Variables	Controls	PD subjects	*P*-value
	**(*n* = 60)**	**(*n* = 58)**	
Gender (Male/Female)	33/27	29/29	0.587
Age (years)	65.82 ± 4.86	65.24 ± 10.18	0.694
Length of hospital stay (day)	N.A.	13 (8∼20)	N.A.
Disease duration (years)	N.A.	3 (1.5∼7.0)	N.A.
Family history [*n*(%)]	N.A.	2 (3.45)	N.A.
Dyslipidemia [*n*(%)]	N.A.	6 (10.34)	N.A.
Diabetes mellitus [*n*(%)]	N.A.	12 (20.69)	N.A.
Hypertension [*n*(%)]	N.A.	34 (58.62)	N.A.
Brain infarction [*n*(%)]	N.A.	15 (25.86)	N.A.
Smoking [*n*(%)]	N.A.	8 (13.79)	N.A.
Drinking [*n*(%)]	N.A.	3 (5.17)	N.A.
SBP/(mmHg)	N.A.	143 (120∼158)	N.A.
DBP/(mmHg)	N.A.	82 (75∼87)	N.A.
H&Y stage	N.A.	2 (1.5∼2.5)	N.A.
ALT/(U/L)	23 (15∼30)	14.5 (6.0∼23.5)	0.001
AST/(U/L)	23 (21∼30)	20.5 (15.0∼30.5)	0.001
ALP/(U/L)	72 (60.5∼83.5)	69 (58.5∼74.0)	0.859
BUN/(mmol/L)	5.41 (4.78∼6.05)	5.11 (4.31∼6.93)	0.019
Cr/(μmol/L)	69.90	70.35	0.221
	(63.35∼81.60)	(60.55∼79.35)	
UA/(μmol/L)	312.65	228.60	0.038
	(250.80∼392.95)	(168.00∼256.70)	
Cys C/(mg/L)	0.93 (0.85∼1.09)	1.03 (0.84∼1.30)	0.002
Glu/(mmol/L)	5.00 (4.77∼5.39)	5.00 (4.85∼5.40)	0.979
TC/(mmol/L)	4.69 (4.06∼5.44)	4.33 (3.64∼4.90)	0.020
TG/(mmol/L)	0.93 (0.78∼1.20)	0.88 (0.65∼1.25)	0.309
HDL/(mmol/L)	1.39 (1.26∼1.67)	1.30 (1.07∼1.60)	0.091
LDL/(mmol/L)	2.87 (2.38∼3.44)	2.60 (1.90∼3.01)	0.013
SAA/(mg/L)	4.20 (2.65∼6.40)	7.0 (4.70∼10.90)	<0.001
HCY/(μmol/L)	12.35 (10.15∼14.10)	14.5 (12.1∼18.7)	<0.001
Lp-PLA2/(ng/mL)	108 (100∼167)	291 (189∼421)	<0.001

*N.A. not available.*

### Comparison of General Data of PD Patients in Different Course of Disease Groups

According to the different courses of the disease, PD patients were divided into a <5 years group and a ≧5 years group. Results showed that the course of disease, H&Y stage and Lp-PLA2 level in the ≧5 years group were all significantly higher than those in the <5 years group (*P* < 0.05). However, the percentage of hypertension in the ≧ 5 years group was significantly lower than that in the <5 years group (*P* < 0.05). In addition, other indicators were not significantly different between the two groups (Table 2).

**TABLE 2:** Analysis of general data of PD patients in different course of disease groups.

Variables	<5 years (*n* = 41)	≥5 years (*n* = 17)	*P*-value
Gender (Male/Female)	22/19	7/10	0.387
Age (years)	66.95 ± 8.72	61.12 ± 12.39	0.069
Length of hospital stay (day)	6.0 (4.5∼12.5)	11.0 (6.5∼12.5)	0.771
Disease duration (years)	3.0 (1.5∼4.0)	5.0 (5.0∼10.0)	<0.001
Family history [*n*(%)]	1 (2.44)	1 (5.88)	0.513
Dyslipidemia [*n*(%)]	6 (14.63)	0	
Diabetes mellitus [*n*(%)]	12 (29.27)	0	
Hypertension [*n*(%)]	28 (68.29)	6 (35.29)	0.020
Brain infarction [*n*(%)]	12 (29.27)	3 (17.65)	0.358
Smoking [*n*(%)]	7 (17.03)	1 (5.88)	0.261
Drinking [*n*(%)]	3 (7.32)	0	
SBP/(mmHg)	127.0 (114.5∼138.5)	120.0 (116.5∼168.5)	0.397
DBP/(mmHg)	85.5 (78∼89.5)	80.0 (75∼87.5)	0.412
H&Y stage	1.75 (1.25∼2.00)	2.50 (2.00∼3.00)	<0.001
ALT/(U/L)	20.5 (13.5∼27.5)	12.0 (6.0∼13.0)	0.765
AST/(U/L)	18 (16.5∼27.5)	18.0 (17.5∼22.0)	0.542
ALP/(U/L)	77.5 (69∼86.5)	65.0 (60.0∼68.0)	0.352
BUN/(mmol/L)	5.39 (4.61∼6.23)	5.16 (4.76∼5.28)	0.048
Cr/(μmol/L)	61.55 (53.80∼78.75)	75.5 (61.3∼86.3)	0.347
UA/(μmol/L)	247.8 (183.9∼307.2)	398.7 (247.2∼408.0)	0.932
Cys C/(mg/L)	0.97 (0.74∼1.02)	1.07 (0.98∼1.16)	0.584
Glu/(mmol/L)	5.70 (5.03∼5.80)	4.62 (4.60∼4.70)	0.069
TC/(mmol/L)	4.34 (3.57∼4.85)	4.31 (3.75∼4.90)	0.590
TG/(mmol/L)	0.88 (0.69∼1.28)	0.77 (0.61∼1.18)	0.463
HDL/(mmol/L)	1.26 (1.07∼1.47)	1.38 (1.17∼1.82)	0.197
LDL/(mmol/L)	2.59 (1.98∼2.94)	2.67 (1.90∼3.01)	0.980
CER/(mg/L)	234.5 (222.0∼255.0)	230.0 (222.0∼255.0)	0.385
SAA/(mg/L)	6.6 (4.7∼10.8)	9.6 (6.7∼15.1)	0.124
HCY/(μmol/L)	14.1 (11.8∼18.4)	15.0 (13.6∼19.7)	0.356
Lp-PLA2/(ng/mL)	162 (110∼229)	320 (314∼458)	0.014

### Comparison of General Data Among PD Patients With Different Stage

According to the different stages of the disease, PD patients were divided into a mild group and a moderate to severe group. Results showed that the course of disease, H&Y stage and Lp-PLA2 in the moderate to severe group were significantly higher than those in the mild group (*P* < 0.05). At the same time, we found that the other indicators of the two groups were not statistically significantly different (Table 3).

**TABLE 3:** Comparison of laboratory findings between mild and moderate to severe outcomes of patients with PD.

Variables	H&Y stage		*P*-value
	Mild group	Moderate to severe	
	(*n* = 41)	group (*n* = 17)	
Gender (Male/Female)	21/20	8/9	0.773
Age (years)	66.39 ± 9.15	62.47 ± 12.18	0.312
Length of hospital stay (day)	10 (5∼13)	9.5 (8∼11)	0.877
Disease duration (years)	2 (1∼4)	5 (5∼9)	<0.001
Family history [*n*(%)]	1 (2.44)	1 (5.88)	0.513
Dyslipidemia [*n*(%)]	5 (12.20)	1 (5.88)	0.472
Diabetes mellitus [*n*(%)]	10 (24.39)	2 (11.76)	0.280
Hypertension [*n*(%)]	24 (58.54)	10 (58.82)	0.984
Brain infarction [*n*(%)]	11 (26.83)	4 (23.53)	0.794
Smoking [*n*(%)]	7 (17.07)	1 (5.88)	0.261
Drinking [*n*(%)]	3 (7.32)	0 (0)	
SBP/(mmHg)	129 (118∼140)	124 (120∼137)	0.817
DBP/(mmHg)	86 (78∼91)	81 (80∼86)	0.694
H&Y stage	1.5 (1∼2)	3 (2.5∼3)	<0.001
ALT/(U/L)	17 (10∼23)	12.5 (8∼15)	0.884
AST/(U/L)	19 (17∼23)	19.5 (18∼21)	0.503
ALP/(U/L)	74 (60∼83)	68.5 (65∼74)	0.918
BUN/(mmol/L)	4.59 (3.89∼5.73)	4.46 (3.55∼5.28)	0.174
Cr/(μmol/L)	65.7 (54.8∼80.7)	64.8 (61.3∼86.2)	0.585
UA/(μmol/L)	258.4 (210∼299.4)	323.7 (246.2∼398.7)	0.494
Cys C/(mg/L)	0.99 (0.86∼1.10)	1.02 (0.90∼1.16)	0.945
Glu/(mmol/L)	5.00 (4.46∼5.57)	4.85 (4.60∼5.27)	0.462
TC/(mmol/L)	4.47 (3.64∼5.02)	4.10 (3.68∼4.83)	0.573
TG/(mmol/L)	0.86 (0.69∼1.22)	1.07 (0.63∼1.25)	0.688
HDL/(mmol/L)	1.29 (1.08∼1.60)	1.34 (1.02∼1.41)	0.952
LDL/(mmol/L)	2.66 (1.95∼2.98)	2.35 (1.90∼3.01)	0.489
CER/(mg/L)	240 (231∼263)	242.5 (222∼292)	0.927
SAA/(mg/L)	7.0 (5.1∼10.8)	7.0 (4.7∼10.9)	0.785
HCY/(μmol/L)	14.4 (10.8∼19.2)	14.6 (13.7∼16.4)	0.785
Lp-PLA2/(ng/mL)	117 (100∼196)	317 (282∼381)	<0.001

### Comparison of General Data Among PD Patients With Different Lp-PLA2 Levels

According to the different levels of serum Lp-PLA2, PD patients were divided into a <200 ng/mL group and a ≧200 ng/mL group. The H&Y stage and Lp-PLA2 level in the ≧200 ng/mL group were significantly higher than in the <200 ng/mL group (*P* < 0.05). However, other indicators were not statistically different between the two groups (Table 4).

**TABLE 4:** Baseline characteristics of patients with PD according to the levels of serum Lp-PLA2.

Variables	Lp-PLA2 (ng/mL)		*P*-value
	<200 (*n* = 29)	≥200 (*n* = 29)	
Gender (Male/Female)	13/16	16/13	0.431
Age (years)	65.83 ± 9.11	64.66 ± 11.28	0.665
Length of hospital stay (day)	10.0 (5.5∼11.0)	9.0 (7.0∼12.5)	0.857
Disease duration (years)	3.0 (0.9∼4.0)	5.0 (2.5∼8.0)	0.122
Family history [*n*(%)]	1 (3.45)	1 (3.45)	1.000
Dyslipidemia [*n*(%)]	5 (17.24)	1 (3.45)	0.085
Diabetes mellitus [*n*(%)]	6 (20.69)	6 (20.69)	1.000
Hypertension [*n*(%)]	17 (58.62)	17 (58.62)	1.000
Brain infarction [*n*(%)]	9 (31.03)	6 (20.69)	0.368
Smoking [*n*(%)]	4 (13.79)	4 (13.79)	1.000
Drinking [*n*(%)]	1 (3.45)	2 (6.90)	0.553
SBP/(mmHg)	125 (117∼135)	134 (121.5∼145.5)	0.237
DBP/(mmHg)	84 (76∼91)	84 (80∼90)	0.901
H&Y stage	1.5 (1.0∼2.0)	2.5 (2.0∼3.0)	<0.001
ALT/(U/L)	17 (10.5∼21.0)	13 (9.0∼20.5)	0.815
AST/(U/L)	20 (16.0∼23.5)	18 (17.5∼22.0)	0.656
ALP/(U/L)	73 (62.5∼83)	69 (60∼79)	0.641
BUN/(mmol/L)	4.42 (3.77∼6.26)	4.76 (3.60∼5.26)	0.446
Cr/(μmol/L)	69.35 (54.80∼85.35)	62 (54.95∼71.90)	0.932
UA/(μmol/L)	266.5 (233.4∼342.3)	258.4 (197.3∼364.0)	0.768
Cys C/(mg/L)	0.99 (0.88∼1.16)	1.03 (0.89∼1.07)	0.503
Glu/(mmol/L)	4.90 (4.43∼5.39)	5.0 (4.61∼5.29)	0.692
TC/(mmol/L)	4.47 (3.74∼4.98)	4.23 (3.63∼4.85)	0.988
TG/(mmol/L)	0.76 (0.61∼1.25)	0.89 (0.74∼1.22)	0.240
HDL/(mmol/L)	1.26 (1.07∼1.66)	1.34 (1.13∼1.46)	0.895
LDL/(mmol/L)	2.67 (1.95∼2.92)	2.49 (1.90∼3.20)	0.988
CER/(mg/L)	243.0 (232.5∼270.0)	234.0 (221.0∼258.5)	0.293
SAA/(mg/L)	7.1 (5.2∼10.8)	6.7 (4.4∼10.9)	0.519
HCY/(μmol/L)	13.6 (10.8∼18.5)	14.6 (13.6∼18.7)	0.437
Lp-PLA2/(ng/mL)	110 (100∼128)	314 (271∼373)	<0.001

### Correlation Analysis Between Lp-PLA2 and Other Indicators

The correlation between Lp-PLA2 and other indicators was analyzed. The Lp-PLA2 level in PD patients was positively correlated with H&Y stage (*P* < 0.01). However, Lp-PLA2 and other indicators showed no significant correlation (*P* > 0.05; Table 5).

**TABLE 5:** Correlation analysis between Lp-PLA2 and other indicators.

Variables	Lp-PLA2		Variables	Lp-PLA2	
	*r*	*P* value		*r*	*P*-value
Age	−0.002	0.988	Disease duration	0.157	0.240
SBP	0.151	0.259	DBP	−0.008	0.952
H&Y stage	0.529	<0.001	UA	0.061	0.648
Cys C	0.048	0.722	TC	−0.005	0.971
TG	0.173	0.193	HDL	0.001	0.993
LDL	−0.003	0.984	SAA	0.037	0.790
HCY	0.013	0.922	ALT	0.115	0.388
AST	0.150	0.260	ALP	−0.024	0.859
GGT	0.114	0.393	BUN	0.186	0.163
Cr	0.150	0.260	Glu	0.005	0.971

### Logistic Regression Analyses of the Risk Factors of PD

Logistic regression analysis was performed to seek the risk factors of PD. Results suggested that HCY, UA, and Lp-PLA2 level were all significantly associated with PD. The levels of HCY and Lp-PLA2 were associated with the risk of PD with odds ratio (OR) of 1.249 [95% confidence interval (CI):1.049–1.488] and 1.008 (95% CI: 1.003–1.014), respectively. However, the UA level was associated with the low risk of PD with OR of 0.993 (95% CI: 0.986–0.999; Table 6).

**TABLE 6:** Logistic regression analysis of the risk factors of PD.

Variable	OR	95% CI	*P*-values	OR*	95% CI	*P*-values
SAA	1.103	0.996∼1.221	0.059	1.098	0.990–1.216	0.076
HCY	1.212	1.030∼1.425	0.020	1.249	1.049–1.488	0.013
UA	0.992	0.986∼0.998	0.005	0.993	0.986–0.999	0.023
Cys C	1.163	0.826∼1.636	0.387	1.365	0.917–2.029	0.125
Lp-PLA2	1.008	1.003∼1.014	0.002	1.008	1.003–1.014	0.004

CI, confidence interval; OR, odds ratio. *Adjusting for age, gender.

### Diagnostic Value of Lp-PLA2 for PD

**FIGURE 1:**
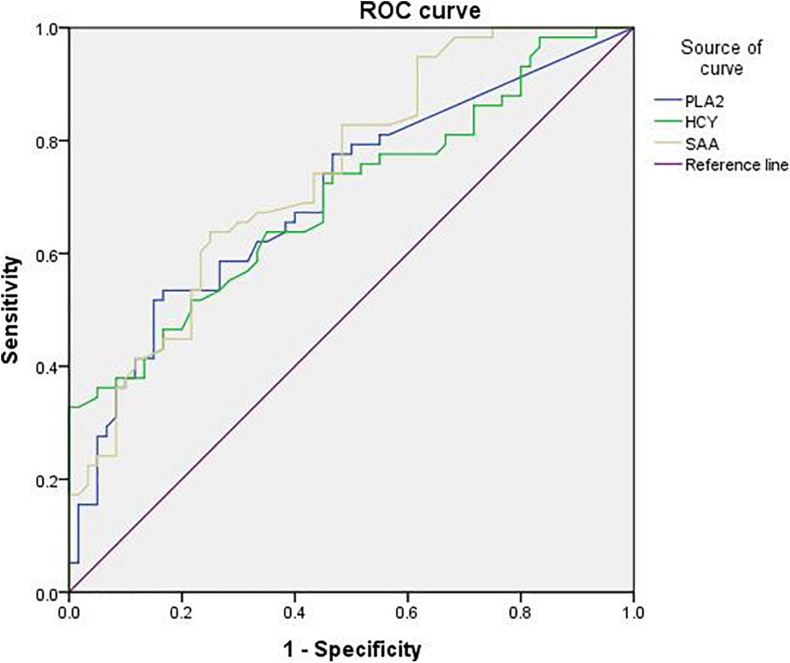
The ROC curve of Lp-PLA2 for discrimination between patients in PD and control group.

The sensitivity of Lp-PLA2 for PD diagnosis was 53.4%, the specificity was 83.3%, and the Youden’s index was 0.367. The sensitivity of SAA for PD diagnosis was 63.8%, the specificity was 75%, and the Youden’s index was 0.388. The sensitivity of HCY for PD diagnosis was 32.8%, the specificity was 100%, and the Youden’s index was 0.328 (Table 7). In addition, the diagnostic value of Lp-PLA2 was between those of HCY and SAA. The ROC curves of Lp-PLA2, SAA, and HCY for discrimination between the PD group and the control group were shown in Figure 1.

**TABLE 7:** Diagnostic value of Lp-PLA2 for PD.

Variable	AUC	95% CI	Sensitivity	Specificity	Youden’s index
Lp-PLA2	0.703	(0.608–0.797)	0.534	0.833	0.367
HCY	0.698	(0.603–0.792)	0.328	1.000	0.328
SAA	0.742	(0.655–0.829)	0.638	0.750	0.388

## Discussion

Lipoprotein-associated phospholipase A2 mainly mediates vascular inflammation by regulating lipid metabolism in the blood. Therefore, although other researchers have conducted extensive research on it, they often only focus on the relationship between Lp-PLA2 and vascular inflammation-related diseases, especially atherosclerosis-related diseases. Lp-PLA2 mainly hydrolyzes oxidized LDL into lysophosphatidylcholine (lysoPC) and oxidized non-esterified fatty acids to play a pro-inflammatory effect (Huang et al., 2020). Lp-PLA2 is considered to be a reliable biomarker of cardiovascular risk, and many reports have identified Lp-PLA2 as an independent predictor of the prognosis of coronary heart disease (Packard, 2009).

Recently, the research focus on Lp-PLA2 has gradually shifted from peripheral vascular inflammatory diseases to cerebrovascular-related diseases. Among them, the most-studied topic is the relationship between Lp-PLA2 and acute cerebral infarction (ACI). Studies confirm that elevated levels of Lp-PLA2 often increase the risk of ACI (Wei et al., 2017; Tao et al., 2020). In addition, reports suggested that Lp-PLA2 may be closely related to the occurrence of AD. Unfortunately, at present, a study on the relationship between Lp-PLA2 and AD has not reached a consensus. Three studies demonstrate no significant correlation between the plasma level or activity of Lp-PLA2 and AD (Davidson et al., 2012; van Himbergen et al., 2012; Savas et al., 2016). Nevertheless, another case-control study shows that higher plasma Lp-PLA2 is independently associated with AD and interacts with cardiovascular diseases, thereby increasing the risk of AD (Doody et al., 2015). Similarly, Fitzpatrick et al. (2014) demonstrate that Lp-PLA2 is an independent risk factor of dementia and AD by comparing the highest and lowest quartiles of Lp-PLA2 level. In view of the sample size of the study and the follow-up time, Lp-PLA2 may be a risk factor of AD, and Lp-PLA2 is involved in the pathogenesis of AD. More importantly, more studies show that Lp-PLA2 is related to the occurrence of cognitive impairment. After using MMSE to evaluate the cognitive ability of patients, early researcheres found that people with high Lp-PLA2 levels had a higher risk of cognitive impairment (van Oijen et al., 2006; Zhu et al., 2019). Although many of the above studies show that Lp-PLA2 is involved in the pathogenesis of certain neurodegenerative diseases, the role of Lp-PLA2 in PD is still unknown. Current researches show that various types of PLA2 are involved in the process of inflammatory response. Among them, one type is cytoplasmic PLA2 (cPLA2), which is also involved in the progression of PD. Recent studies found that when a selective PLA2 inhibitor is applied to the SH-SY5Y cellular model, while the intracellular oligomerization and monomericα-syn were drastically reduced, cell survival increased compared with before, which means that cPLA2 inhibitors can reduce the deposition of potentially pathological α-syn nucleoprotein in cells (Xylaki et al., 2020). Based on this, we speculate that PLA2 activity participates in the occurrence and development of PD through the regulation of neuronal α-syn aggregation. In another study, Seet et al. (2010) in the detection of plasma lipid and DNA oxidation products in 61 PD patients, found that compared with the control group, the plasma PLA2 and PAF-AH activities of PD patients were reduced, which led to an accumulation of esterified F2-isoprostanes in the lipids and participated in the onset and progression of PD. As far as we know, our study is the first to explore the relationship between Lp-PLA2 and PD.

Lipoprotein-associated phospholipase A2 level is higher in PD patients, and the increase in its level is related to the risk of PD. We first compared the demographic and clinical characteristics between PD patients and the controls. We found that in addition to Lp-PLA2 level, the expression levels of Cys C, SAA, and HCY were higher in the PD patients than those of the control group. Our findings on the relationships between the latter three indicators and PD are consistent with other epidemiological and clinical studies (Song et al., 2013; Li et al., 2020). At the same time, compared with controls, PD patients had lower levels of ALT, AST, TC, LDL, BUN, and UA in the serum. The decrease of UA level led to the decrease of antioxidant capacity in PD patients, which was in line with current research (Sakuta et al., 2017). In terms of disease courses, the serum Lp-PLA2 level and H&Y stage of PD patients with a longer course were significantly higher than those with a shorter course. In addition, after using H&Y to stage PD patients, we found that PD patients with milder conditions had significantly lower serum Lp-PLA levels and disease duration than patients with more severe conditions. Moreover, logistic regression analysis showed that both Lp-PLA2 and HCY were independent risk factors of PD, while UA was a protective factor of PD. We further used ROC curves to analyze the diagnostic characteristics of Lp-PLA2, HCY, and SAA for PD, and found that the diagnostic value of Lp-PLA2 was between those of HCY and SAA, which provide a reference value for the combined use of multiple indicators.

Although our findings indicate that Lp-PLA2 may be a risk factor of PD, the mechanism of Lp-PLA2 in PD is still unclear. Doody RS et al. did not detect the Lp-PLA2 expression in the brain tissues of AD patients and controls. The involvement of Lp-PLA2 in the pathogenesis of AD may be related to its vascular damage (Doody et al., 2015). However, we do not know whether Lp-PLA2 is expressed in PD brain tissues. Considering the damaging effect of Lp-PLA2 on endothelial cells, we speculate that Lp-PLA2 is also involved in the pathogenesis of PD through vasculitis damage. As reported, lysoPC can induce pericytes loss in the CNS, which is indicative of an injured BBB (Muramatsu et al., 2015). The BBB precisely regulates the material exchange between the blood and the brain to maintain the microenvironment homeostasis of the CNS. The destruction of the BBB in PD has been confirmed by many studies. The breakdown of the BBB due to the destruction of tight junctions, inflammation or other risk factors may increase the permeability of BBB and cause the penetration of inflammatory cells, inflammatory factors and other harmful substances from blood vessels to the brain, thereby participating in the PD process (Desai et al., 2007; Erdo et al., 2017). Studies show that Lp-PLA2 and its main enzyme product lysoPC can participate in diabetic retinopathy by damaging the blood-retinal barrier (BRB; Canning et al., 2016; Acharya et al., 2017). Based on the structural similarity between BRB and BBB, we believe that Lp-PLA2 can damage the BBB and participate in the PD process. Reportedly, darapladib, an inhibitor of Lp-PLA2, can reduce the permeability of BBB (Acharya et al., 2013). However, whether darapladib has a significant effect on PD and whether Lp-PLA2 can be used as a potential therapeutic target for PD need to be further studied.

Although our research shows that Lp-PLA2 is related to the risk of PD, it still has some limitations. Firstly, we need to enlarge the sample size of PD patients, especially the PD patients with longer disease course and more serious disease, and further confirm the relationship between Lp-PLA2 and the risk of PD. Secondly, we do not consider the use of lipid-lowering drugs. Some PD patients may have taken oral lipid-lowering drugs, which can lead to a decrease in LP-PLA2 levels to some extent, but does not affect our conclusions as a whole. Thirdly, in our study, some PD patients have a history of hypertension and cerebral infarction, which may have a certain degree of influence on the level of Lp-PLA2, which may be considered as a potential bias. Finally, we only detect the mass of Lp-PLA2, but not its activity. In addition, although more and more studies have shown that elevated levels of Lp-PLA2 is a high-risk underlying factor for cardiovascular diseases, an authoritative randomized controlled clinical trial study shows that in patients with stable coronary heart disease, the Lp-PLA2 inhibitor darapladib did not significantly reduce the probability of cardiovascular death, myocardial infarction or stroke in such patients (White et al., 2014). Based on this speculation, whether Lp-PLA2 can become our expected target for the treatment of PD may still require a lot of scientific research to verify. Whether the activity of Lp-PLA2 is related to the risk of PD remains to be further revealed.

## Conclusion

In conclusion, the increased level of Lp-PLA2 is associated with the risk of PD. This biomarker is related to vascular inflammation, suggesting a potential mechanism for PD-related destruction of the BBB. In view of the convenience and safety of serum Lp-PLA2 monitoring, it can be used for early detection of PD and may provide a potential intervention target for clinical treatment of PD.

## Data Availability Statement

The original contributions presented in the study are included in the article/supplementary material, further inquiries can be directed to the corresponding author/s.

## Ethics Statement

The studies involving human participants were reviewed and approved by Tongji Medical College, Huazhong University of Science and Technology. The patients/participants provided their written informed consent to participate in this study.

## Author Contributions

ZW and TL designed the study, participated in the experiment, conducted the statistical analysis, and wrote the manuscript. LW contributed to the completion of the final manuscript. SW collected the data. All authors contributed to the article and approved the submitted version.

## Conflict of Interest

The authors declare that the research was conducted in the absence of any commercial or financial relationships that could be construed as a potential conflict of interest.
